# SCALING UP A FALL PREVENTION PROGRAM IN RURAL COMMUNITIES: FEASIBILITY AND LESSONS LEARNED

**DOI:** 10.1093/geroni/igac059.2598

**Published:** 2022-12-20

**Authors:** Melissa O'Connor, Jane Strommen, Sean Brotherson, Philip Estepp, Rachel Grace, Megan Pedersen, Teri Undem, Divya Saxena

**Affiliations:** North Dakota State University, Moorhead, Minnesota, United States; North Dakota State University, Fargo, North Dakota, United States; North Dakota State University, Fargo, North Dakota, United States; North Dakota State University, Fargo, North Dakota, United States; North Dakota State University, Fargo, North Dakota, United States; North Dakota State University, Fargo, North Dakota, United States; North Dakota State University, Fargo, North Dakota, United States; North Dakota State University, Fargo, North Dakota, United States

## Abstract

In North Dakota, 28% of adults over age 65 report falling each year, resulting in substantial morbidity and mortality. Evidence-based fall prevention programs can benefit quality of life and maintenance of daily living activities, particularly for community-dwelling older adults. The Stepping On fall prevention program, a national evidence-based program, is designed to educate older adults about risk factors for falls, safety strategies, and coping behaviors. The program consists of seven weekly community-based workshops conducted in a small-group setting by two trained facilitators. The Stepping On program was introduced in North Dakota in March 2012. Since that time, 132 workshops have taken place in rural communities across the state, serving 1,502 participants. The number of workshops and participants steadily grew between 2012 and 2019. In the year 2012-13, 127 individuals participated in ten workshops. By 2019, before the program was suspended during the pandemic, 199 individuals participated in 16 workshops; 24 workshops had previously been held in 2018. The current study discusses the implementation process as the program scaled up and expanded to more locations. The feasibility and sustainability of conducting the program in isolated rural areas, challenges encountered, and lessons learned will also be discussed. It is important that residents in rural communities have access to evidence-based programs like Stepping On so they can experience the benefits.

